# Renin as a Marker of Tissue Perfusion, Septic Shock and Mortality in Septic Patients: A Prospective Observational Study

**DOI:** 10.3390/ijms23169133

**Published:** 2022-08-15

**Authors:** Patrycja Leśnik, Lidia Łysenko, Małgorzata Krzystek-Korpacka, Ewa Woźnica-Niesobska, Magdalena Mierzchała-Pasierb, Jarosław Janc

**Affiliations:** 1 Department of Anaesthesiology and Intensive Therapy, 4th Military Clinical Hospital, 50-981 Wroclaw, Poland; 2 Department of Anaesthesiology and Intensive Therapy, Wroclaw Medical University, 50-556 Wroclaw, Poland; 3 Department of Biochemistry and Immunochemistry, Wroclaw Medical University, 50-369 Wroclaw, Poland

**Keywords:** sepsis, septic shock, renin-angiotensin-aldosterone system, lactate concentration, survival marker, mortality rate

## Abstract

Sepsis is a life-threatening organ dysfunction caused by the dysregulation of the host’s response to an infection, where the dominant mechanism is tissue hypoperfusion. Currently, the marker used to define tissue disorders is lactate levels, which may be elevated in other disease states as well. Renin is an essential hormone for the proper functioning of the renin-angiotensin-aldosterone (RASS) system. It is secreted in the glomerular apparatus in response to hypoperfusion. This study aimed to assess the usefulness of renin as a marker of tissue hypoperfusion in patients with sepsis and septic shock. A final group of 48 patients treated for sepsis and septic shock in the intensive care unit was included. Blood samples for renin quantification were collected in the morning as a part of routine blood analysis on the first, third, and fifth days. Sepsis was diagnosed in 19 patients (39.6%), and septic shock was diagnosed in 29 patients (60.4%). There was no significant difference in renin concentration between patients who received and did not receive continuous renal replacement therapy (CRRT) on any study day. Therefore, all samples were analyzed together in subsequent analyses. There was a significant difference in renin concentration between sepsis survivors and non-survivors on the third (31.5 and 119.9 pg/mL, respectively) and fifth (18.2 and 106.7 pg/mL, respectively) days. As a survival marker, renin was characterized by 69% and 71% overall accuracy if determined on the third and fifth days, respectively. There was a significant difference in renin concentration between sepsis and septic shock patients on the first (45.8 and 103.4 pg/mL, respectively) and third (24.7 and 102.1 pg/mL, respectively) days. At an optimal cut-off of 87 pg/mL, renin had very good specificity and a positive likelihood ratio. Renin was a strong predictor of mortality in patients with sepsis and septic shock. Further, the level of renin in patients with septic shock was significantly higher than in patients with sepsis. In combination with the assessment of lactate concentration, renin seems to be the optimal parameter for monitoring tissue hypoperfusion and could be helpful for septic shock diagnosis, as well as for identifying candidate patients for CRRT.

## 1. Introduction

Sepsis is an inflammatory response to infection characterized by multiple organ dysfunctions and ultimately death. It is defined as a life-threating organ dysfunction caused by the dysregulation of the host’s response to an infection [1]. Sepsis is estimated to affect more than 30 million people worldwide every year [2]. The mortality risks of sepsis range between 15% and 56%, potentially leading to 6 million deaths [3,4,5,6,7]. It remains one of the key causes of health loss worldwide, although the rates of age-adjusted incidence have decreased over the last three decades [8].

The activation of the renin-angiotensin-aldosterone system (RAAS), a multi-organ endocrine system designated to prevent systemic hypotension under hypovolemic conditions [9], is part of a physiological response to shock [10]. Circulating RAAS hormones are responsible for blood pressure, as well as fluid and ion homeostasis, whereas the local RAAS is associated with inflammation, apoptosis, cellular growth, and vascular permeability [11]. The RAAS is activated by blood loss or a drop in blood pressure, stimulating juxtaglomerular cells to initiate proteolytic activation of prorenin. The juxtaglomerular cells sense changes in renal perfusion pressure via stretch receptors in the vascular walls. The primary function of the resulting renin is to increase blood pressure, leading to the restoration of perfusion pressure in the kidneys. Renin is released into the circulation and converts liver-synthesized angiotensinogen into a decapeptide angiotensin I, which is subsequently cleaved by an angiotensin-converting enzyme (ACE) to form a biologically active octapeptide angiotensin II (ANG II). ANG II is a potent vasoconstrictor, as well as a pro-fibrotic and proinflammatory agent that interacts with its type I receptor (AT_1_R). However, its binding to the less abundant type II receptor (AT_2_R) has an opposing effect. The hormone increases blood pressure by increasing its volume via several mechanisms, including acting on the hypothalamus to induce thirst, on the kidneys to release antidiuretic vasopressin, and on the adrenal gland to release aldosterone, a steroid hormone that induces sodium retention in the kidneys and other organs. Several other biologically active peptides belonging to the RAAS family, (alternatively cleaved or substituted with different amino acids) as well as receptors, have recently been recognized [10,12].

The sepsis-associated deregulation of RAAS frequently manifests as increased activity of plasma renin and increased concentrations of angiotensin I (ANG I) and ANG II, but with concurrent low aldosterone [10]. Even when elevated, ANG II often does not exert the expected effect, likely due to the decreased expression of angiotensin receptors [13,14]. Up to 30% of critically ill patients have hyperreninemic hypoaldosteronism, which is associated with acute renal failure, organ dysfunction, and death [15]. Plasma aldosterone is a useful biomarker for indicating which patients have a high risk of renal dysfunction [15,16,17,18]. Renin, in turn, is advantageous as a marker of hypoperfusion and is considered a good index for RAAS activity [9]. Renin has a molecular mass of 37 kDa and displays diurnal variations in healthy patients. It is not removed by standard hemofiltration, and its production could be affected by medication (e.g., by beta-agonists, RAAS blockers, and furosemide) [19]. A detailed understanding of the dysfunctions of the renin-angiotensin-aldosterone system may give rise to new treatment options targeting specific disorders within the RAAS.

This study was designed to determine renin’s potential as a new marker of tissue hypoperfusion in a representative population of critically ill patients, and to assess its value as a septic shock and survival marker compared to other available parameters.

## 2. Results

### 2.1. Patients’ Characteristics

**Figure 1:**
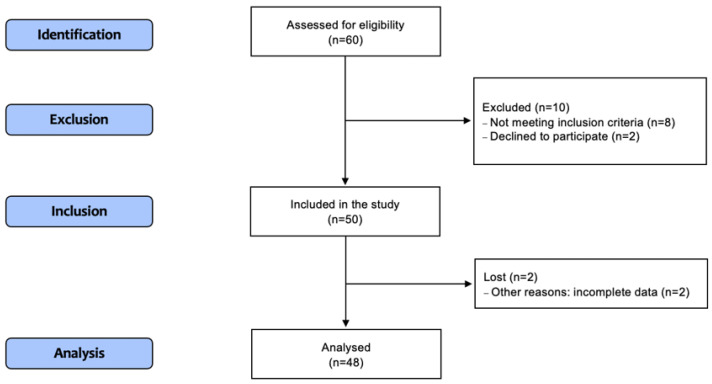
STROBE flow chart of the study participants.

During the study period between September 2015 and April 2019, 80 patients were admitted to the ICU and assessed for eligibility. Of these, 48 patients were included in the study. A flow chart of the patients’ selection is shown in Figure 1. Sepsis was diagnosed in 19 patients (39.6%), and septic shock was diagnosed in 29 patients (60.4%). The most common sources of sepsis were pneumonia (39.6%) and abdominal infections (35.4%). The characteristics of patients, stratified based on condition (sepsis and septic shock) and survival, are shown in Table 1.

### 2.2. Removal of Renin by Continuous Renal Replacement Therapy (CRRT)

**Figure 2:**
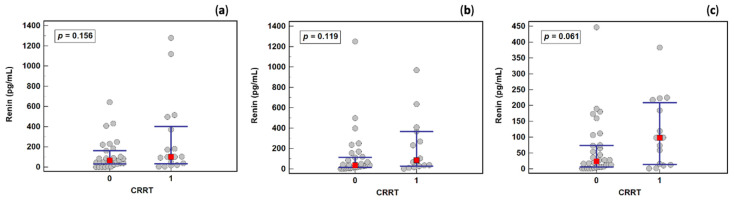
The effect of continuous renal replacement therapy (CRRT) on renin concentration: (**a**) on the first study day; (**b**) the third study day; (**c**) in the fifth study day. Notes: Data were analyzed using Mann–Whitney *U* tests, and are presented as medians (red squares) with interquartile ranges (blue whiskers).

There was no significant difference in renin concentration between patients who received and did not receive CRRT on any study day (Figure 2). Therefore, all samples were analyzed together in subsequent analyses.

### 2.3. Renin Concentration during Follow-Up

**Figure 3:**
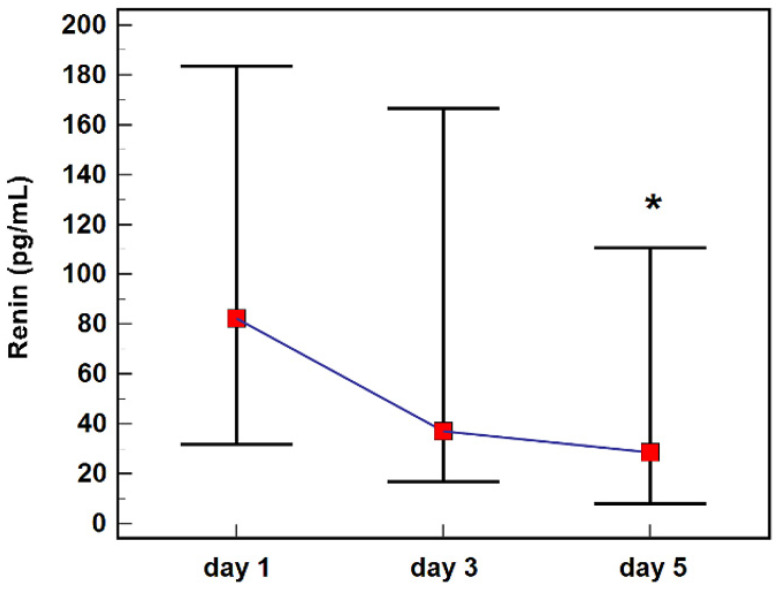
Renin concentration during a follow-up. Notes: Data analyzed using Friedman tests, and are presented as medians (red squares) with interquartile ranges (black whiskers). *, significantly (*p* < 0.05) different from measurements at day 1 and 3.

The highest renin concentration was observed on the first day after diagnosis (82.3 pg/mL) and its level gradually decreased on the third and fifth days of observation (37.1 pg/mL and 28.4 pg/mL, respectively), differing significantly at fifth day compared to first and third days (Figure 3).

### 2.4. Renin Association with Biochemical and Clinical Parameters and Follow-Up Treatment

Renin concentration positively correlated with liver parameters, specifically the activity of aminotransferases on the first, and bilirubin concentration on the fifth study days. On the first day, renin also positively correlated with hematocrit and hemoglobin concentrations, and on the fifth day, it positively correlated with inflammatory markers (CRP and leukocytes), and inversely correlated with urea and pH. Renin concentration was consistently and significantly associated with lactate and MAP on the first and fifth days, while the association with CVP was significant solely on the third study day. Regarding sepsis clinical scores, renin concentration positively correlated solely with SOFA on the third and fifth study days (Table 2). There was also a positive correlation between renin concentration and the noradrenaline dose administered on the third (ρ = 0.37, *p* = 0.014) and fifth (ρ = 0.36, *p* = 0.022) study days.

### 2.5. Renin as a Septic Shock Marker

**Figure 4:**
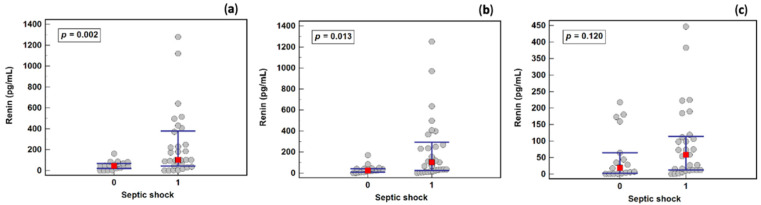
The effect of septic shock on renin concentration: (**a**) on the first study day; (**b**) on the third study day; (**c**) on the fifth study day. Legend: 0—sepsis group, 1—septic shock group. Notes: Data were analyzed using Mann– Whitney *U* tests, and are presented as medians (red squares) with interquartile ranges (blue whiskers).

There was a significant difference in renin concentration between sepsis and septic shock patients on the first (45.8 and 103.4 pg/mL, respectively) and third (24.7 and 102.1 pg/mL, respectively) days, but not on the fifth day (18.9 and 58.6 pg/mL, respectively) (Figure 4).

The ROC curve analysis (Table 3) indicated that renin (determined on the first day), as a predictor, was characterized by moderate overall accuracy, but a good Youden index. At an optimal cut-off of 87 pg/mL, renin had very good specificity and a positive likelihood ratio.

The ROC curve analysis (Table 3) indicated that lactate, APACHE, and SOFA were superior shock markers in terms of overall accuracy, but only lactate was superior to renin in terms of the Youden index and positive likelihood ratios.

### 2.6. Effect of Septic Shock on Renin Dynamics

**Figure 5:**
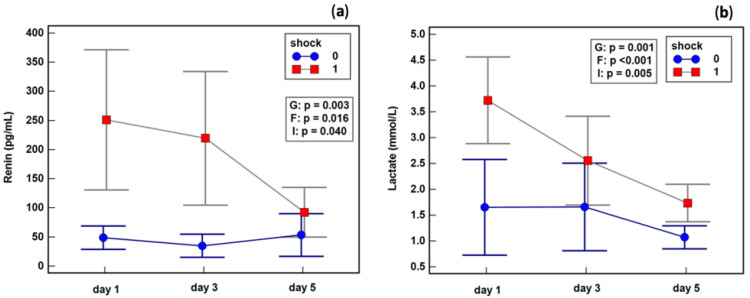
Effect of septic shock on the dynamics of (**a**) renin; (**b**) lactate. Legend: 0—patients without septic shock, 1—patients with septic shock. Notes: Data were analyzed using repeated measures ANOVAs, and are presented as means (red squares and blue dots) with 95% confidence intervals (gray and blue whiskers). G, the group effect (survivors vs. non-survivors); F, the factor effect (day of testing); I, group and factor interaction.

The changes in renin concentration during follow-up were dependent on the presence of shock. Septic shock patients experienced a decrease in renin concentration between the third and fifth study days, while patients without shock exhibited a slight increase. Shock patients experienced a drop in lactate between the first and third days, whereas its concentration in patients without shock remained stable (Figure 5).

### 2.7. Renin as a Survival Marker

**Figure 6:**
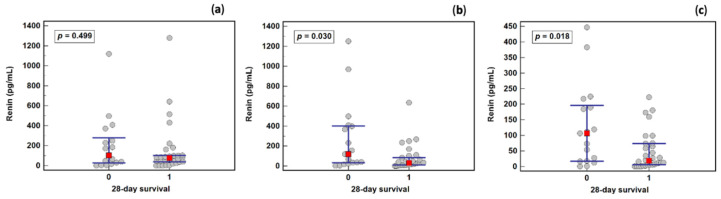
Renin concentration in sepsis survivors and non-survivors: (**a**) on the first study day; (**b**) the third study day; (**c**) in the fifth study day. Legend: 0—non-survivors, 1—survivors. Notes: Data were analyzed using Mann–Whitney *U* tests, and are presented as medians (red squares) with interquartile ranges (blue whiskers).

There was a significant difference in renin concentration between sepsis survivors and non-survivors on the third (31.5 and 119.9 pg/mL, respectively) and fifth (18.2 and 106.7 pg/mL, respectively) days, but not on the first day (75.1 and 102.1 pg/mL, respectively) (Figure 6).

**Figure 7:**
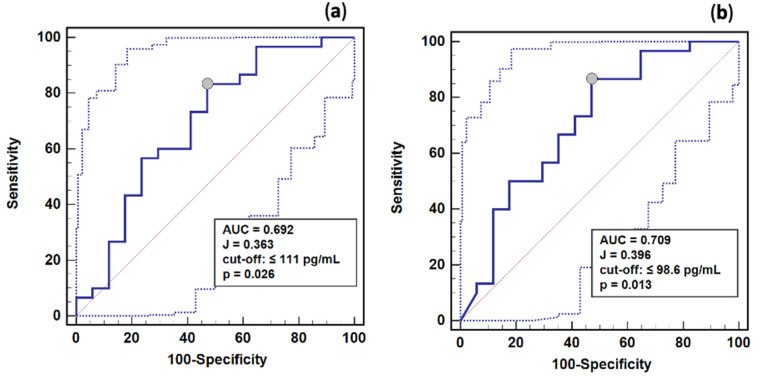
Renin as a 28-day survival marker (blue line): (**a**) determined on the third study day; (**b**) determined on the fifth study day. Legend: Dotted lines—95% CI. Notes: Data were analyzed using receiver operating characteristics (ROC) curve analysis. AUC, area under ROC curve, interpreted as marker’s overall accuracy; J, Youden index.

**Figure 8:**
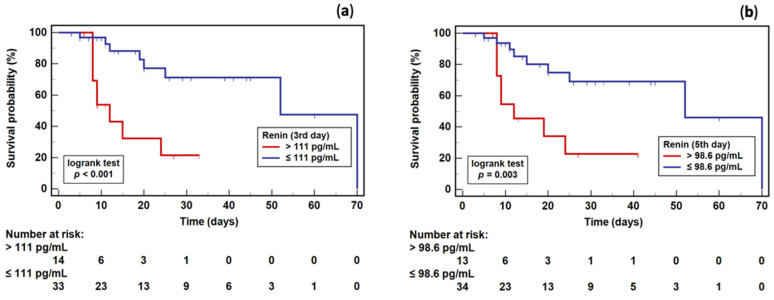
The Kaplan–Meier survival curves for septic patients stratified based on renin concentration: (**a**) assessed on the third day; (**b**) assessed on the fifth day.

As a survival marker, renin was characterized by 69 and 71% overall accuracy if determined on the third and fifth days, respectively (Figure 7). Using optimal cut-off values for renin, as determined in the ROC curve analysis, patients were stratified into groups, and a survival analysis was conducted. Patients with a renin concentration ≤111 pg/mL and ≤98.6 pg/mL on the third and fifth day, respectively, had a significantly higher survival probability than those with a higher renin concentration (Figure 8). The relative risk of death in patients with renin >111 pg/mL on the third day was 8.7% (95%CI: 2.5–29.7) higher, and with renin >98.6 pg/mL on the fifth day was 6.4% (95%CI: 1.8–28.9) higher than in patients with renin ≤111 or 98.6 pg/mL, respectively.

### 2.8. Renin Dynamics in Sepsis Survivors and Non-Survivors

**Figure 9:**
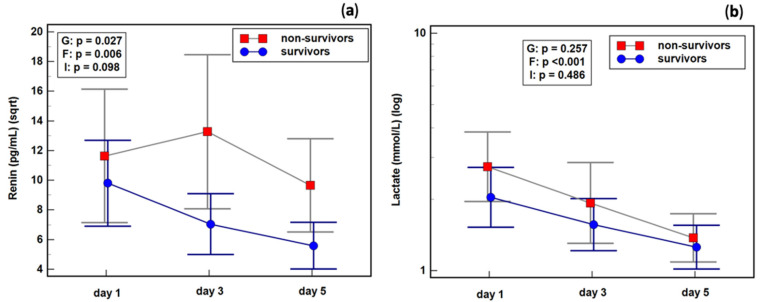
Renin and lactate dynamics in sepsis survivors and non-survivors: (**a**) renin; (**b**) lactate. Notes: Data were analyzed using repeated measures ANOVAs on data transformed into squared roots (sqrt; renin) or logarithms (log; lactate) and are presented as means (red squares and blue dots) with 95% confidence intervals (gray and blue whiskers). G, the group effect (survivors vs. non-survivors); F, the factor effect (day of testing); I, group and factor interaction.

The changes in renin concentration during follow-up were affected by the patients’ survival. Renin increased between the first and third days in non-survivors, but dropped in survivors. The changes in lactate were comparable between survivors and non-survivors (Figure 9).

## 3. Discussion

In this study, we attempted to evaluate the usefulness of renin, besides lactate levels, as an additional biomarker of hypoperfusion in the course of sepsis and septic shock. The usefulness of renin with a cut-off of 87 pg/mL as a marker of the development of septic shock was demonstrated. At the same time, no effect of renal replacement therapy on blood renin concentration was demonstrated, which makes it a universal marker in patients treated in the ICU.

Sepsis is an inflammatory response to infection, and multiple organ failures contribute to the mortality of afflicted patients. Early restoration of systemic oxygen delivery aids in the resuscitation of patients with septic shock, but in contrast to other forms of shock, microvascular perturbations persist despite optimized global hemodynamics [20]. Because disturbed microvasculature results in diminished nutrient extraction [21], clinicians now search for therapeutic goals for microvascular resuscitation in severe cases of sepsis [22]. According to the Surviving Sepsis Campaign: International Guidelines for Management of Sepsis and Septic Shock 2021 [23], one of the main criteria for the diagnosis of septic shock is the level of lactate ≥2 mmoL/L, despite volume resuscitation. However, the use of lactate as a marker of hypoperfusion is limited. Its increased level may be due to reasons other than from organ perfusion disorders in the course of sepsis and septic shock. In situations in which cells cannot be provided with an adequate supply of oxygen, their only source of energy is the process of anaerobic glycolysis. The accumulated pyruvic acid is then converted into lactic acid by lactate dehydrogenase (LDH) [11,24,25]. In the case of cardiogenic shock, aortic thrombosis, epileptic seizures, muscle tremors, renal liver failure, decompensated diabetes, neoplastic disease, intoxication with ethanol, glycol, insulin, morphine, salicylates, and other drugs, an increase in lactate levels is observed [25]. Further, after extensive surgical procedures, such as aortic prosthetic reconstruction, liver transplantation, and restoration of peripheral arterial vessels, patients may experience increased anaerobic metabolism due to significant oxygen consumption, which would lead to an increase in lactate levels.

Lactate elevation is one of the criteria to diagnose septic shock, and is a result of, and thus a marker for, tissue hypoxia [21]. Accordingly, in our cohort, lactate outperformed renin, a tissue hypoperfusion marker, as well as SOFA and APACHE II scores, as a septic shock indicator when assessed on the first day. On the third day, however, renin, and lactate were comparable as septic shock markers.

Both elevated lactate levels and decreased clearance have been predictive of mortality [21]. In the present study, however, lactate concentrations did not differ significantly between survivors and non-survivors, in contrast to renin.

Renin is a 40 kDa glycoprotein produced in the glomerular apparatus of the afferent arteriole. When the adrenergic system is activated, the amount of sodium reaching the dense macula decreases, or the perfusion pressure in the glomerular vessels decreases, which then stimulates renin secretion. In the event of a drop in blood pressure, a feedback mechanism is activated due to the release of renin into the blood in greater amounts, which causes an increase in system pressure and ensures proper blood flow through the kidneys. The only site of renin secretion is in the kidneys; therefore, only disturbances in blood flow through the kidneys can lead to its secretion. Renin testing is now a simple test and is available in most hospitals.

Our study showed both a significant difference in renin concentration between sepsis and septic shock patients on the first (45.8 and 103.4 pg/mL, respectively) and third (24.7 and 102.1 pg/mL, respectively) days, and a significant difference in renin concentration between sepsis survivors and non-survivors on the third (31.5 and 119.9 pg/mL, respectively) and fifth (18.2 and 106.7 pg/mL, respectively) days of treatment. It seems that the application of an additional criterion, which could be a renin value above 87 pg/mL and lactates above 2 mmol/L, would allow for a confirmation of the correct diagnosis of septic shock in cases where there is doubt about the source of the elevated lactate concentration.

The changes in renin concentration during follow-up were affected by the patients’ survival. Renin increased between the first and third days in non-survivors, but dropped in survivors, whereas the changes in lactate were comparable between survivors and non-survivors. Similar observations were shown in the work by Gleeson et al. [14], in which the level of renin was strongly correlated with mortality.

Khanna et al. [19] in the Critical Care Editorial suggests that despite the fact that renin is not a marker of impaired microcirculation, it can act as a predictor of tissue hypoperfusion and improvement of tissue blood flow, and thus, can be a good predictor of mortality in sepsis. Chung et al. [22] also showed that plasma renin activity is a strong predictor of mortality in sepsis.

Plasma renin activity and ANG II concentrations correlate with impairments in microvascular dysfunction, organ failure, and mortality. Plasma renin activity has not been found to correlate with mean arterial pressure (MAP), preventing the claim that arterial pressure is the main contributor to activated RAAS in sepsis [26].

Unlike in mice, the activity of the RAAS system in humans is dependent on the availability of renin. In this study, consistent with Kurtz’s [24] study, and with norepinephrine being a stimulatory clue for renin secretion, we showed that renin concentration is positively correlated with norepinephrine dose in our patients. Disturbances in microcirculatory blood flow are associated with elevated central venous pressure (CVP), an indicator of hemodynamic status, and a mortality predictor in adult [27] and pediatric septic shock patients [25]. Although renin correlated positively with CVP on the third day, CVP was neither a septic shock nor a mortality predictor in our cohort.

## 4. Materials and Methods

### 4.1. Study Patients

The study group consisted of 48 patients hospitalized in the intensive care unit (ICU) of the Department of Anesthesiology and Intensive Therapy of Wroclaw Medical University between 2014 and 2019. The study design included prospective blood sampling and retrospective data analysis. All patients met the criteria for sepsis and septic shock based on the current guidelines by the Surviving Sepsis Campaign (SSC): International Guidelines for Management of Sepsis and Septic Shock 2021 [23]. Exclusion criteria included patients with severe immunosuppression (cancer, chronic steroid therapy, or chemotherapy), or patients < 18 years old.

### 4.2. Study Design

Demographic, clinical, and biochemical data at the time of ICU admission were recorded. The Acute Physiology and Chronic Health Evaluation (APACHE) II and the Sequential Organ Failure Assessment (SOFA) scores were calculated at the initial time of ICU admission. The SOFA score was also recorded on the first, third, fifth, and seventh day. Data on the following parameters were collected: age, sex, total fluid input and output, serum creatinine, urea concentration, procalcitonin, C-reactive protein (CRP), lactate, electrolytes, morphology, arterial and venous blood gas analysis, and parameters assessing the coagulation system as well as the liver. Additionally, patients were assessed with respect to their need for mechanical ventilation, ventilator-free days, and/or renal replacement therapy, type of hemofilter, and dosage of catecholamines and fluids.

### 4.3. Blood Sampling and Renin Determination

Blood samples for biomarker quantification were collected in the morning as a part of routine blood analysis on the first, third, and fifth days. Blood was drawn by arterial line and collected into serum-separator tubes, clotted (20 min, room temperature), and centrifuged (10 min, 720× *g*). Serum aliquots were stored at −80 °C until analysis. Renin concentration was measured in duplicates with a commercially available enzyme-linked immunosorbent assay–Renin (active) ELISA (IBL International GmbH, Hamburg, Germany), according to the manufacturer’s instructions. The test sensitivity was 0.81 pg/mL. The median intra-assay coefficient of variation (%CV) was 2.0, and an inter-assay %CV was 3.2.

### 4.4. Ethical Considerations

The study protocol was approved by the Bioethics Committee of Wroclaw Medical University in Poland (permission Nos. KB-548/2014 and KB-670/2017). The study was carried out in accordance with the guidelines of the Declaration of Helsinki and Good Clinical Practice. Informed and written consent was obtained from all patients. The Strengthening the Reporting of Observational Studies in Epidemiology (STROBE) guidelines were followed, and the flow chart is shown in Figure 1.

### 4.5. Statistical Analysis

Statistical analysis was performed using MedCalc^®^ version 20.014 (MedCalc Software Ltd., Ostend, Belgium). The distribution of all the quantitative parameters was checked for consistency with the normal distribution. The conformity assessment was carried out using the Kolmogorov–Smirnov test. Homogeneity of variances was tested using the Levene’s test. The critical significance level was assumed at *p* < 0.05. Nominal and ordinal data were presented as a contingency table, and analyzed using the Chi-squared or exact Fisher test. Quantitative data were presented as means with standard deviations or medians with lower and upper quartiles. Normally distributed data with homogeneous variances were analyzed using *t*-tests for independent samples, while non-normally distributed data, or data with non-homogenous variances, were analyzed using Mann–Whitney *U* tests. Due to a non-normal data distribution, renin concentration during the follow-up was analyzed using the Friedman test. To analyze the difference in renin and lactate dynamics with respect to the presence of shock or patients’ survival, repeated measures ANOVAs were run, and data on renin and lactate were transformed prior to analysis into squared roots or logarithms, respectively. Correlation analyses were conducted using Spearman rank correlations (ρ). Diagnostic potential was assessed using a receiver operating characteristics (ROC) curve analysis and expressed in terms of area under the ROC curve (AUC), which was interpreted as a marker’s overall accuracy. In addition, a marker’s sensitivity, specificity, and likelihood ratios (LR), corresponding with an optimal cut-off (the cut-off at which the Youden index was the highest) were calculated. The Kaplan–Meier and log-rank test were used to determine renin association with survival.

## 5. Conclusions

Renin is a strong predictor of mortality in patients with sepsis and septic shock. The levels of renin in patients with septic shock were significantly higher than those in patients with sepsis. It seems that in combination with the assessment of lactate concentration, renin may be an optimal parameter for monitoring tissue hypoperfusion and helpful for septic shock diagnosis, as well as in patients receiving renal replacement therapy.

## Figures and Tables

**Table 1:** Characteristics of study population.

Characteristics	All Patients	Condition/Subtype		Survival	
		Sepsis	Septic Shock	Survivors	Non-Survivors
*N*	48	19	29	31	17
Sex, F/M (%)	19/29	5/14	14/15 ^1^	14/17	5/12 ^2^
Age (years), Me (IQR)	65 (54.3; 71.5)	69 (51.3–73.5)	63.5 (55–70) ^1^	63 (54.3–70)	66 (54.5–73) ^2^
APACHE II, Me (IQR)	23.5 (16.5; 29)	16 (13.3–21)	26 (22–30) ^3^	19 (15–28.5)	25 (22.8–29.3) ^2^
SOFA, Me (IQR)	10 (8; 13)	8 (6.3–10.8)	12 (9.8–14) ^3^	10 (7–12.8)	11 (9–14) ^2^
GCS, Me (IQR)	14.5 (10.5; 15)	15 (13.3–15)	13 (8–15) ^3^	15 (13–15)	12 (8–13.5) ^4^
HGB (g/dL), Me (IQR)	10.4 (9.7; 12.1)	10.1 (8.3; 10.7)	10.6 (9.9; 12.6) ^3^	10.4 (9.2; 12.3)	10.2 (9.9; 11.9) ^2^
HCT (%), Me (IQR)	32 (28.5; 36.4)	30.6 (25.4; 31.8)	33 (30; 40) ^3^	31.3 (27.6; 38.4)	32.2 (30.1; 35.3) ^2^
WBC (×10^3^/µL), M ± SD	15.5 ± 10.4	14.97 ± 9.2	15.88 ± 11.3 ^1^	14.66 ± 9.7	17.08 ± 11.8 ^2^
PLT (×10^3^/µL), M ± SD	228 ± 107	253 ±112	212 ± 103 ^1^	243 ± 106	200 ± 107 ^2^
INR, Me (IQR)	1.241 (1.11; 1.45)	1.2 (1.07; 1.28)	1.32 (1.12; 1.6) ^1^	1.211 (1.12; 1.47)	1.286 (1.17; 1.41) ^2^
D-dim. (µg/mL), Me (IQR)	6.1 (4.3; 13.7)	8.55 (4.6; 13.8)	5.58 (4.1; 13.7) ^1^	7.68 (4; 14.7)	5.55 (4.9; 13.1) ^2^
SaO_2_ (%), Me (IQR)	97.8 (95.3; 99)	98.4 (97.5; 99.2)	96.2 (92; 98.8) ^3^	97.6 (95.1; 99)	98 (95.6; 99.1) ^2^
PaO_2_ (mmHg), Me (IQR)	111 (88; 133)	126 (107; 175)	84 (67; 135) ^3^	111 (71; 145)	111 (81; 162) ^2^
PaCO_2_ (mmHg), Me (IQR)	38.7 (35.9; 45.4)	38.4 (36.6; 42.4)	38.8 (30.8; 45.8) ^1^	39 (36.7; 45.5)	37.8 (29.9; 44) ^2^
PaO_2_/FiO_2_, M ± SD	245.8 ± 151.5	273.5 ± 155.3	224.2 ± 148.2 ^1^	233.2 ± 155.8	270.2 ± 145.2 ^2^
BE (mM), M ± SD	−3.61 ± 5.9	−3.28 ± 4.7	−3.83 ± 6.6 ^1^	−3.16 ± 5.9	−4.42 ± 5.9 ^2^
HCO_3_^-^ (mM), Me (IQR)	21.5 (17.6; 25.3)	21.8 (19; 25.5)	20.9 (17.3; 24) ^1^	21.8 (19; 25.2)	20.8 (16.7; 26.5) ^2^
Lactate, Me (IQR)	1.95 (1.4; 4.0)	1.3 (0.9; 1.6)	2.7 (2.1; 5.1) ^3^	1.7 (1.2; 4.1)	2.4 (1.9; 3.8) ^2^
GLU (mg%), Me (IQR)	145 (118; 194)	130 (109; 149)	150 (124; 207) ^3^	146 (115; 202)	140 (118; 182) ^2^
Urea (mg/dL), Me (IQR)	77 (48; 120)	57 (42; 87)	88.5 (58.5; 146.5) ^3^	75 (52; 107)	85 (45; 132) ^2^
CREAT (mg/dL), Me (IQR)	1.64 (0.85; 2.35)	1.03 (0.74; 2.19)	1.76 (1.17; 2.47) ^1^	1.72 (0.8; 2.55)	1.39 (0.9; 2.3) ^2^
DIUR (mL/day), Me (IQR)	1140 (330; 230)	1095 (913; 1910)	1185 (100; 2375) ^1^	1095 (379; 1968)	1710 (80; 2145) ^2^
BIL (mg/dL), Me (IQR)	0.9 (0.6; 1.5)	0.9 (0.6; 1.3)	0.9 (0.5; 1.6) ^1^	0.9 (0.6; 1.4)	0.95 (0.6; 1.8) ^2^
ASPAT (U/L), Me (IQR)	64 (27.5; 218)	27 (14.5; 74.8)	128 (47.5; 354) ^3^	56 (39.5; 279)	100 (24; 183) ^2^
ALAT (U/L), Me (IQR)	58 (20.8; 83.5)	27 (12; 63)	63 (37.5; 221) ^3^	59 (13.8; 99.5)	58 (46; 73) ^2^
CRP (mg/L), M ± SD	238.4 ± 119.4	261 ± 116.4	221.9 ± 121.1 ^1^	240.2 ± 110.3	234.4 ± 141.9 ^2^
PCT (ng/mL), Me (IQR)	6.43 (2.44; 43.1)	3 (1.3; 21.7)	8.4 (4.4; 55.5) ^1^	5.3 (2.4; 18.3)	22.8 (3; 55) ^2^
MAP (mmHg), Me (IQR)	95.3 (78.8; 126.9)	93.3 (82.4; 107)	109 (70; 197) ^1^	95 (77.2; 112)	107.4 (78.8; 166) ^2^
CVP (mmHg), M ± SD	7 ± 3.1	7.7 ± 3.3	6.4 ± 2.9 ^1^	7.3 ± 2.8	6.6 ± 3.7 ^2^
Source of sepsis, *n* (%):					
IAI	19 (39.6)	11 (57.9)	8 (27.6) ^1^	13 (41.9)	6 (35.3) ^2^
CNS	3 (6.3)	1 (5.3)	2 (6.9)	0	3 (17.6)
PNEU	19 (39.6)	6 (31.6)	13 (44.8)	12 (38.7)	7 (41.2)
UTI	4 (8.3)	1 (5.3)	3 (10.3)	4 (12.9)	0
SSTI	2 (4.2)	0	2 (6.9)	1 (3.2)	1 (5.9)
BSI	1 (2.1)	0	1 (3.5)	1 (3.22)	0
CRRT, *n* (%):					
Day 1	17 (35.4)	3 (15.8)	14 (48.3) ^3^	10 (32.3)	7 (41.2) ^2^
Day 3	14 (29.2)	2 (10.5)	12 (41.4) ^3^	8 (25.8)	6 (35.3) ^2^
Day 5	15 (31.2)	2 (10.5)	13 (44.8) ^3^	29 (29)	6 (35.3) ^2^

Notes: ^1^, *p* > 0.05 as compared to sepsis; ^2^, *p* > 0.05 as compared to survivors; ^3^, *p* ≤ 0.05 as compared to sepsis; ^4^, *p* ≤ 0.05 as compared to survivors. Abbreviations: *N*, number of patients; F/M, female-to-male ratio; Me, median; IQR, interquartile range; M, arithmetic mean; SD, standard deviation; APACHE II, the Acute Physiology and Chronic Health Evaluation II score; SOFA, the Sequential Organ Failure Assessment score; GCS, the Glasgow coma scale; HGB, hemoglobin; HCT, hematocrit; WBC, white blood cell count; PLT, thrombocyte count; INR, an international normalized ratio (prothrombin time); D-dim., D-dimers; SaO_2_, arterial oxygen saturation; PaO_2_, arterial oxygen partial pressure; PaCO_2_, arterial carbon dioxide partial pressure; PaO_2_/FiO_2_; arterial oxygen partial pressure-to-fractional inspired oxygen ratio; BE, base excess; GLU, glucose; CREAT, creatinine; DIUR, diuresis; BIL, bilirubin; ASPAT; aspartate aminotransferase; ALAT, alanine aminotransferase; CRP, C-reactive protein; PCT, procalcitonin; MAP, mean arterial pressure; CVP, central venous pressure. IAI, intrabdominal infection; CNS, central nervous system; PNEU, pneumoniae; UTI, urinary tract infection; SSTI, soft skin tissue infection; BSI, blood stream infection; CRRT, continuous renal replacement therapy.

**Table 2:** Correlation between renin and biochemical and clinical parameters during a follow-up.

Parameter	Renin		
	1st Day	3rd Day	5th Day
ASPAT	0.36 ^2^	0.13 ^1^	0 ^1^
ALAT	0.34 ^2^	0.26 ^1^	0.17 ^1^
Bilirubin	0.10 ^1^	0.28 ^1^	0.35 ^2^
CRP	−0.08 ^1^	0.13 ^1^	0.33 ^2^
WBC	0 ^1^	0 ^1^	0.40 ^3^
CVP	0.19 ^1^	0.34 ^2^	0.22 ^1^
pH	0.13 ^1^	−0.16 ^1^	−0.32 ^2^
Lactate	0.33 ^2^	0.28 ^1^	0.34 ^2^
Urea	−0.03 ^1^	−0.10 ^1^	−0.29 ^2^
HCT	0.36 ^2^	−0.18 ^1^	0 ^1^
HGB	0.32 ^2^	−0.15 ^1^	0.10 ^1^
SOFA	0.02 ^1^	0.31 ^2^	0.35 ^2^

Notes: Data presented as Spearman correlation coefficients, rho (ρ). Only associations found significant on at least one study day are included. ^1^ *p* > 0.05; ^2^ *p* ≤ 0.05; ^3^ *p* < 0.01. Abbreviations: ASPAT, aspartate aminotransferase; ALAT, alanine aminotransferase; CRP, C-reactive protein; WBC, white blood cells count; CVP, central venous pressure; MAP, mean arterial pressure; HCT, hematocrit; HGB, hemoglobin; SOFA, the Sequential Organ Failure Assessment score.

**Table 3:** Renin and other parameters determined on the first day as septic shock markers.

Parameter	AUC (95% CI), *p*	Cut-Off	J	Sens. %	Spec. %	LR+	LR−
Renin	0.770 (0.62–0.88), *p* < 0.0001	>87 pg/mL	0.634	69	94.4	12.4	0.33
SOFA	0.787 (0.65–0.89), *p* < 0.0001	>8	0.528	89.7	63.2	2.4	0.16
APACHE II	0.815 (0.68–0.91), *p* < 0.0001	>21	0.583	79.3	79	3.8	0.26
Lactate	0.904 (0.78–0.97), *p* < 0.0001	>1.8 mmol/L	0.809	86.2	94.7	16.4	0.15

Abbreviations: AUC, area under receiver operating characteristics (ROC) curve, interpreted as marker’s overall accuracy; J, Youden index; Sens., sensitivity; Spec., specificity; LR, likelihood ratios; SOFA, the Sequential Organ Failure Assessment score; APACHE II, the Acute Physiology and Chronic Health Evaluation II score.

## Data Availability

The data presented in this study are available on request from the corresponding author.
